# Bone mineral density and microarchitecture change during skeletal growth in harbor seals (*Phoca vitulina*) from the German coast

**DOI:** 10.1038/s41598-023-33911-8

**Published:** 2023-05-03

**Authors:** Julian Delsmann, Britta Schmidt, Ralf Oheim, Michael Amling, Tim Rolvien, Ursula Siebert

**Affiliations:** 1 Department of Osteology and Biomechanics, University Medical Center Hamburg-Eppendorf, Lottestr. 59, 22529 Hamburg, Germany; 2 Division of Orthopedics, Department of Trauma and Orthopedic Surgery, University Medical Center Hamburg-Eppendorf, Martinistr. 52, 20246 Hamburg, Germany; 3 Institute for Terrestrial and Aquatic Wildlife Research, University of Veterinary Medicine Hannover, Foundation, Werftstr. 6, 25746 Büsum, Germany

**Keywords:** Ocean sciences, Marine biology

## Abstract

Across species, the skeletal system shares mutual functions, including the protection of inner organs, structural basis for locomotion, and acting as an endocrine organ, thus being of pivotal importance for survival. However, insights into skeletal characteristics of marine mammals are limited, especially in the growing skeleton. Harbor seals (*Phoca vitulina*) are common marine mammals in the North and Baltic Seas and are suitable indicators of the condition of their ecosystem. Here, we analyzed whole-body areal bone mineral density (aBMD) by dual-energy X-ray absorptiometry (DXA) and lumbar vertebrae by high-resolution peripheral quantitative computed tomography (HR-pQCT) in neonate, juvenile, and adult harbor seals. Along skeletal growth, an increase in two-dimensional aBMD by DXA was paralleled by three-dimensional volumetric BMD by HR-pQCT, which could be attributed to an increasing trabecular thickness while trabecular number remained constant. Strong associations were observed between body dimensions (weight and length) and aBMD and trabecular microarchitecture (R^2^ = 0.71–0.92, all p < 0.001). To validate the results of the DXA measurement (i.e., the standard method used worldwide to diagnose osteoporosis in humans), we performed linear regression analyses with the three-dimensional measurements from the HR-pQCT method, which revealed strong associations between the two imaging techniques (e.g., aBMD and Tb.Th: R^2^ = 0.96, p < 0.0001). Taken together, our findings highlight the importance of systematic skeletal investigations in marine mammals during growth, illustrating the high accuracy of DXA in this context. Regardless of the limited sample size, the observed trabecular thickening is likely to represent a distinct pattern of vertebral bone maturation. As differences in nutritional status, among other factors, are likely to affect skeletal health, it appears essential to routinely perform skeletal assessments in marine mammals. Placing the results in the context of environmental exposures may allow effective measures to protect their populations.

## Introduction

Harbor seals (*Phoca vitulina*) are common marine mammals along the German coast and are found in the North and Baltic Seas. As they are apex predators and long-living marine mammals (20–25 years for males and 30–35 years for females), and have a small home range, they are a suitable indicator of the condition of their ecosystem^1–3^. Multiple studies report on different anthropogenic impacts and their effects on the immune, endocrine, and reproduction system of harbor seals in the North and Baltic Sea^2,4–6^. However, only a limited number of studies have investigated the skeletal system and potential changes due to various stressors^7,8^.

Despite fundamental environmental differences, such as between sea and land, the skeletal system shares similar functions across species. At least five shared characteristics of the skeleton can be defined, including providing the structural basis for locomotion, protection of inner organs, the main storage for calcium, bone as an endocrine organ, and sound transmission during hearing^9,10^. Bone is remodeled lifelong through a balance of bone formation and resorption, which is tightly coupled and carried out by two different bone cell types, namely bone-forming osteoblasts and bone-resorbing osteoclasts^11^. Next to these two cell types (i.e., osteoblasts and osteoclasts), matrix-embedded mechanosensitive osteocytes represent the most abundant cell type, orchestrating the remodeling process by various processes, including the production of signaling proteins and hormones^12,13^. Specific alterations in osteocyte characteristics, namely a lower number of osteocyte lacunae per bone area as a lower lacunar area, have previously been shown for sperm whales in comparison to other whale species, as deep diving may be associated with reduced osteocyte viability^14^. The remodeling process is very sensitive to environmental changes and, for this reason, also influenced by multiple factors, such as vitamins, hormones, nutrients, and pollutants, among others^15–17^. Besides, disturbances or alterations in bone quality can have significant impacts on the overall health or physiology of an individual^18^.

In human medicine, bone densitometry is used for diagnostics and treatment monitoring of bone diseases in routine clinical practice, with dual-energy X-ray absorptiometry (DXA) representing the gold standard method^19^. Areal bone mineral density (aBMD) is used in humans for the definition of osteoporosis and assessment of fracture risk^20^. However, a relevant proportion of individuals with fragility fractures characteristic of osteoporosis are not identified by DXA, but by evaluation of bone microarchitecture using high-resolution peripheral quantitative computed tomography (HR-pQCT)^21^. HR-pQCT has been developed as an in vivo successor to micro-CT^22^ to circumvent its analysis capability limited to smaller ex vivo specimens and has since demonstrated substantial added value in routine clinical practice and skeletal research^23^. In this sense, three-dimensional HR-pQCT analyses allow the assessment of bone microarchitecture parameters such as trabecular thickness (Tb.Th) or three-dimensional trabecular bone mineral density (Tb.BMD), which was previously limited to ex vivo evaluations such as bone histomorphometry or quantitative backscattered electron imaging (qBEI).

Wildlife studies on the skeleton are comparably limited, but in the last years, an increase in the number of reports has been noticeable^8,15,24,25^. One marine species is the common bottlenose dolphin (*Tursiops truncatus*), measuring aBMD at different ages in this dolphin^17,23^. The authors showed that aBMD of common bottlenose dolphins increases with age, although a considerable variance of aBMD was found^23^. DXA has been mainly used to measure aBMD in different animal species and investigate distinct bone conditions^15,26,27^, as this method is available worldwide and has excellent validity. Such investigations are important, as multiple different environmental and anthropogenic stressors (e.g., pollutants or overfishing) influence the health status of marine mammals. A study of ringed seals (*Pusa hispida*) demonstrated critical effects of contaminants on aBMD^14^. Marine mammals are at the end of the food chain and therefore can also indicate possible health effects for humans. Additionally, marine mammals are key species for their ecosystems, because they play an important role in structuring the ecosystem they live in. These combined aspects make them good health indicators^1,28^. The skeleton, in particular, can be analyzed to derive relevant information about growth, blood production, reproduction, and mineral and energy storage^29^. In that sense, previous bone examinations have shown critical information about individuals, such as decreased bone mass or pathological changes like increased porosity in east Greenland polar bears, baltic gray seals, and Florida pumas^27,30–32^ or even traumatic alterations such as fractures in harbor porpoises^33^. However, the diagnosis of osteoporosis has not yet been standardized in marine mammals (including harbor seals). Therefore, it appears of pivotal importance to transfer those technologies to wildlife research and conservation.

As the skeleton undergoes a remarkable development during growth, including rapid growth in the first years of the life of mammals^34^, defining age-specific characteristics of harbor seals may be of central relevance. Whereas one study investigated age-associated changes in the microarchitecture of the mandible of harbor seals on the German coast^8^, no reports of vertebral aBMD or bone microarchitecture parameters are available. As the spine is of importance for the functional integrity of the skeleton and thus the survival of the animal, in this study we examined nine harbor seals of three different age groups using DXA and HR-pQCT to determine the skeletal features and their association with age.

## Material and methods

### Samples and necropsy

**Figure 1:**
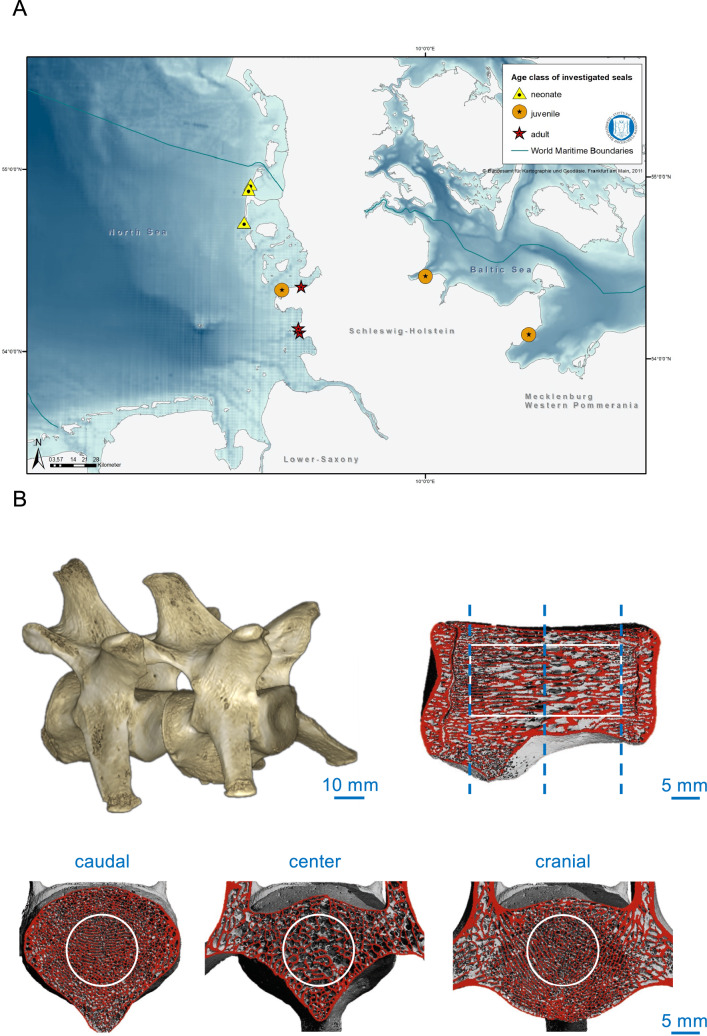
Map of the investigated harbor seals and imaging methodology. (**A**) The triangle shows the neonates, the circles show the juveniles, and the stars are the adult harbor seals. The map was created using ArcGIS software (ArcMap Version 10.6.1). (**B**) Three-dimensional reconstruction of segmented lumbar vertebrae (upper left panel) scanned by HR-pQCT and a sagittal view to indicate the scanned volume of interest (VOI, contoured in white).

The samples originate from harbor seals, which were found dead, bycaught or euthanized by hunters at the German coasts due to conditions incompatible with survival, including completely apathetic, mild to severe cough and breathing sounds, sometimes bloody nose (Fig. 1A). Five of the nine investigated harbor seals were found dead, while the other mammals had to be euthanized (Table 1). To prevent sex bias on skeletal parameters^15^, only female animals were included. A sample of nine harbor seals was examined each three for one age group. Length and body weight including blubber were measured in the animals and their nutritional status was determined based on blubber thickness, measured at the neck (dorsal and ventral) and on the state of the muscles^35,36^. In that sense, it was defined as poor (5–20 mm), moderate (21–30 mm), or good (31–50 mm). This way, they were classified as neonates (≤ 8 weeks of postnatal age), juveniles (8 weeks–5 years of age) or adults (beyond 5 years of age). The necropsies of seals based on the international guidelines for cetacean^36^. Seven of the nine animals were collected from the North Sea, two from the Baltic Sea. All animals were collected between 2018 and 2021. The animals were found all year round, including breeding and molting season. The exact age of five animals was determined by the growth layers counted in the cementum of the canine teeth as described in Lockyer et al.^37^, as animals younger than 1.5 years do not show easily the growth layers in the cementum. The age determination of neonate harbor seals was based on external characteristics including the time of the year it was found and the known time of birth. The classification of the age classes (neonate, juvenile and adult) based on body length^36^. No living animals were used in this study.

**Table 1:** Demographic data of the investigated harbor seals (*Phoca vitulina*) from the German coast.

Animal number	Age group	Age	Water	Date	Nutritional status	Cause of death
*P.v. 1*	Neo	1–2 mo	North Sea	02.08.2019	Poor	Cachexia
*P.v. 2*	Neo	1–2 mo	North Sea	18.08.2019	Poor	Cachexia
*P.v. 3*	Neo	1–2 mo	North Sea	02.08.2019	Poor	Cachexia
*P.v. 4*	Juv	< 1 year	North Sea	14.02.2019	Moderate	Bronchopneumonia with endoparasitosis
*P.v. 5*	Juv	n.d	Baltic Sea	12.06.2020	Good	n.d
*P.v. 6*	Juv	3 years	Baltic Sea	04.02.2021	Moderate	n.d
*P.v. 7*	Adu	17 years	North Sea	30.12.2018	Good	Abortion with a uterine rupture and internal bleeding
*P.v. 8*	Adu	11 years	North Sea	25.05.2020	Good	Intestinal displacement
*P.v. 9*	Adu	17 years	North Sea	04.02.2020	Good	Gastritis with a high grade parasitosis of the stomach

*Neo.* neonate, *mo.* months, *Juv.* juvenile, *Adu.* adult, *n.d.* not determinable. Poor: blubber thickness 5–20 mm; moderate: blubber thickness 21–30 mm; good: blubber thickness 31–50 mm.

### Areal bone mineral density (aBMD)

To evaluate aBMD, dual-energy X-ray absorptiometry (DXA, Lunar Prodigy iDXA; GE Healthcare, Madison, WI, USA) was performed. The DXA device was daily calibrated using a phantom provided by the manufacturer to ensure scan validity. For the analysis, the entire animal was placed on the table of the scanner, and a whole-body scan was conducted. Subsequently, aBMD was calculated using the manufacturer’s software (enCORE-software v15-GE Healthcare Lunar, Buckinghamshire, UK), which determined the BMD in grams per square centimeter (g/cm^2^)^15^. Each scan was checked manually for correct bone segmentation. Total body aBMD values were used for subsequent analyses.

### High-resolution peripheral quantitative computed tomography (HR-pQCT)

After necropsy^35^, the lumbar vertebrae were fixed in 3.7% formaldehyde. For the assessment of the trabecular bone microarchitecture, HR‐pQCT measurements (XtremeCT II^®^, Scanco Medical AG, Brüttisellen, Switzerland) were performed of the entire vertebra using an ex vivo protocol provided from the manufacturer (60 kVp, 900 µA, 100 ms integration time, voxel size of 42 µm, Fig. 1B). Data consistency were ensured by the daily use of the calibration phantom. During the scan, the specimens were fixed within special casts. Within a standardized cylindric volume of interest (VOI) in-between the epiphyses of the vertebrae (Fig. 1B), a three-dimensional microarchitectural dataset was generated and trabecular parameters calculated, including trabecular volumetric bone mineral density (Tb.BMD, mgHA/cm^3^), bone volume per tissue volume (BV/TV), trabecular thickness (Tb.Th, mm), and trabecular number (Tb.N, 1/mm)^23^.

### Statistical analysis

Statistical analysis was performed using GraphPad Prism (version 8.4.0, GraphPad Software, Inc., La Jolla, CA, USA). Normality-distribution of the data were tested using Shapiro–Wilk test. As all data were normally distributed, one-way ANOVA and repeated measures with Tukey correction was carried out for the comparison of the three groups. For the analysis of an association between DXA values, body length or weight and bone microarchitectural parameters, linear regression analyses were performed and the coefficient of determination R^2^ and the regression slopes with confidence intervals (CIs) were calculated. Results are given as absolute values or the mean ± standard deviation (SD). The dashed lines of the truncated violin plots represent the median and quartiles. The level of significance was defined as p < 0.05. Exact p-values are reported unless p < 0.0001.

### Ethic statement

The Institute for terrestrial and aquatic wildlife research (TAW) has been authorized by MEKUN (Ministry for Energy Transition, Climate Protection, Environment and Nature) to perform health monitoring for dead stranded or stranded animals and has all the necessary permits required for this.

## Results

The nutritional status varied among the mammals. All neonates were diagnosed with emaciation (i.e., poor nutritional status), while the nutritional status was classified as moderate in two of the three young animals and good in the remaining animals. All neonate harbor seals died because of cachexia due to separation from the mother. One of the juvenile mammals had a bronchopneumonia with endoparasitosis, whereas for the other two juveniles the reason of death could not be clearly defined (Table 1). The three adults died due to different diseases: One animal had a gastritis with a high grade parasitosis of the stomach, one animal was found with an intestinal displacement and the last one died due to an abortion with a uterine rupture and internal bleeding, causing a sepsis and an associated shock.

**Figure 2:**
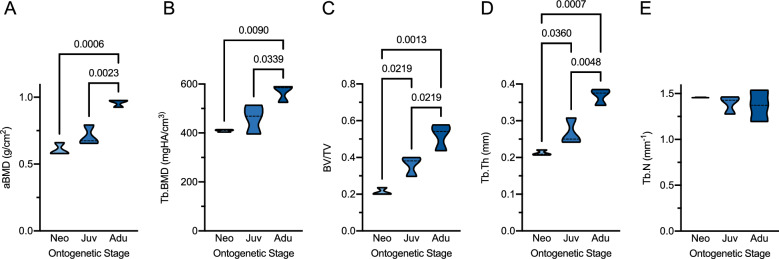
Skeletal parameters between the different age-groups. (**A**) DXA-derived areal bone mineral density (aBMD) and (**B-E**) HR-pQCT-derived trabecular bone microarchitecture including (**B**) trabecular BMD (Tb.BMD), (**C**) bone volume per total volume (BV/TV), (**D**) trabecular thickness (Tb.Th), and (**E**) trabecular number (Tb.N). Only significant p-values (< 0.05) are given for the pairwise comparisons. *Neo* neonate, *Juv* juvenile, *Adu* adult.

For the evaluation of skeletal characteristics, we evaluated the DXA measurements by age group. Notably, an increase in aBMD with age was observed with significant differences between adults and neonates as well as between adults and juveniles but not between neonates and juveniles (Fig. 2A). Likewise, HR-pQCT-derived Tb.BMD was significantly higher in adults compared to neonates and juveniles (Fig. 2B). When analyzing three-dimensional bone microarchitecture parameters, significant differences of BV/TV (Fig. 2C) were observed between each of the age groups, which could be attributed to an increase in Tb.Th (Fig. 2D). No significant differences were observed for Tb.N between the age groups (p = 0.247, Fig. 2E).

**Figure 3:**
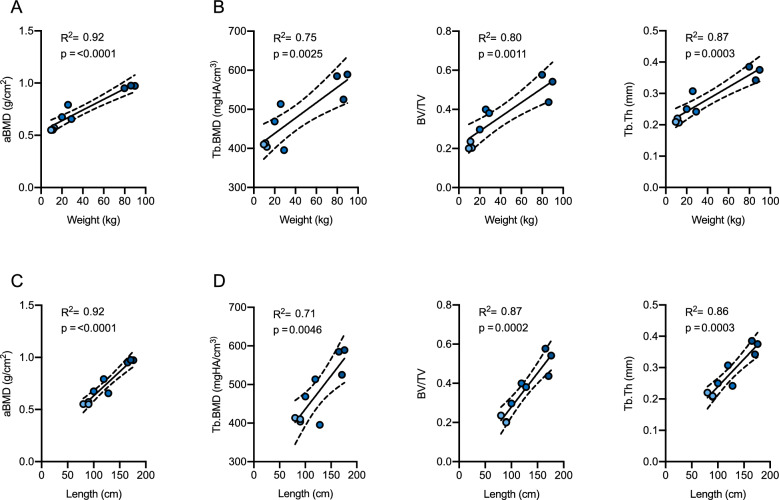
Association between body weight or length and skeletal parameters. Linear regression analysis of body weight and (**A**) DXA-derived areal bone mineral density (aBMD) and (**B**) HR-pQCT-derived trabecular bone microarchitecture including trabecular BMD (Tb.BMD), bone volume per total volume (BV/TV) and trabecular thickness (Tb.Th). Linear regression analysis of body length and (**C**) aBMD and (**D**) trabecular bone microarchitecture.

When evaluating the association between weight and densitometric or microarchitectural parameters, strong associations were observed for all parameters (Fig. 3A,B). Specifically, the strongest regressions were shown between body weight and aBMD as well as Tb.Th (R^2^ = 0.92, p < 0.0001 and R^2^ = 0.87, p = 0.0003, respectively). Comparable strong associations were detected between body length and aBMD as BV/TV, whereas three-dimensional Tb.BMD showed moderate to strong associations with body weight (R^2^ = 0.75, p = 0.003) and length (R^2^ = 0.71, p = 0.005, Fig. 3C,D). Interestingly, no association with body weight and length was observed for Tb.N (R^2^ = 0.028, p = 0.669 and R^2^ = 0.099, p = 0.408, respectively).

**Figure 4:**
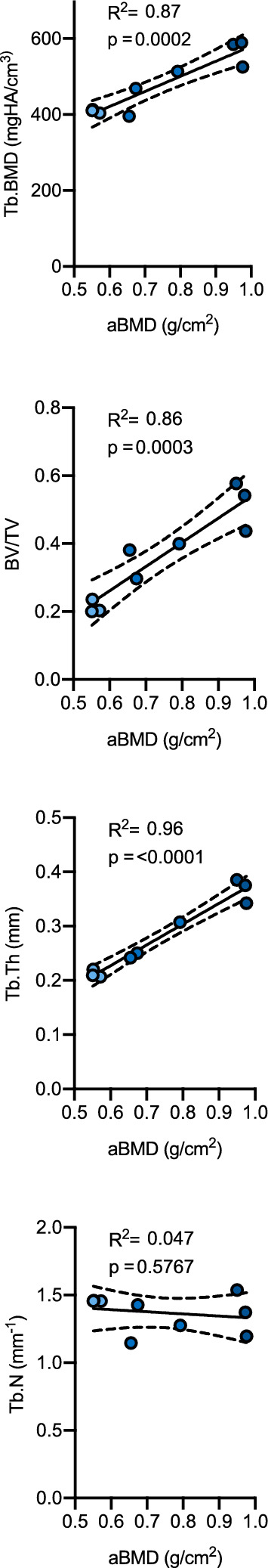
Association between skeletal parameters derived from DXA and HR-pQCT measurements. *aBMD* areal bone mineral density, *Tb.BMD* trabecular bone mineral density, *BV/TV* bone volume per total volume, *Tb.Th* trabecular thickness, *Tb.N* trabecular number.

Lastly, as HR-pQCT analysis is not as globally available as DXA, we aimed to compare the findings of DXA and HR-pQCT measurements to answer the question of transferability for clinical routine. Of note, strong positive associations between two-dimensional aBMD and most of the three-dimensional bone microarchitecture parameters were observed between the two imaging modalities (Fig. 4). In that sense, aBMD and volumetric BMD (i.e., Tb.BMD) showed strong associations (R^2^ = 0.87, p = 0.0002), comparable to BV/TV (R^2^ = 0.86, p = 0.0003), which was surpassed by Tb.Th (R^2^ = 0.96, p < 0.0001). Interestingly, no association was observed between aBMD and Tb.N, which remained stable regardless of the increase in aBMD (R^2^ = 0.047, p = 0.577).

## Discussion

Despite the importance of skeletal health for the survival of marine mammals, insights into the characteristics of bone mineral density and especially bone microarchitecture during growth are scarce. As harbor seals (*Phoca vitulina*) are common along the German coast with a comparably long lifespan, they have been used as a suitable indicator for the respective ecosystem before^2^. The findings of the necropsies showed different conditions leading to death in the nine harbor seals, varying between the age groups as nutritional status^36^. All identified causes of death found are occurring frequently in this species in German waters and are therefore representative findings.

As the spine is of pivotal importance for the functional integrity of the skeleton and thus the survival of the animal^38^, extending the knowledge of the individual properties in different species exposed to varying environmental conditions and ways of locomotion appears of great scientific and clinical relevance. Previously, in-depth skeletal examinations of specific anatomic sites in harbor seals were limited to the temporomandibular joint^39,40^. Specifically, one report using HR-pQCT showed an increase in cortical thickness but a decrease in BV/TV and Tb.Th during growth for a portion of the mandibular corpus^8^. However, little is known about age-related changes in vertebral aBMD and the microarchitecture of harbor seals. Assessment of vertebral aBMD corresponds to the clinical standard site in human medicine and might be the most appropriate place to make relevant deductions such as fracture risk. Moreover, previous studies in marine mammals investigating aBMD characteristics focused predominantly on the effects of environmental pollutants^17,32^ but not on age-group associated changes.

With this first study of its kind in harbor seals, we were able to show that, except for Tb.N, vertebral parameters of aBMD and bone microarchitecture change with ontogenetic skeletal growth. These findings suggest that the basis for the osseous scaffold or skeleton is developed at a very young age, marked by a comparably high number of trabeculae, becoming increasingly mineralized and increasing in thickness as development progresses. In this regard, thickening of the trabeculae was also observed in human children with increasing age^41^. However, it must be noted that both DXA and HR-pQCT are not able to detect unmineralized bone matrix (i.e., osteoid), likely resulting in low structural parameters at a young age.

Because it is accepted that lower mechanical load is associated with a decline in skeletal characteristics^42^ and, especially, cachexia secondary to poor nutritional status is likely to affect bone mineral density^43,44^, the measured parameters of such neonates are prone to be conditionally inferior compared to harbor seals of the same age but in good nutritional status. This also applies, to a smaller degree, to the juvenile seals, of whom two showed a moderate nutritional status. Therefore, future studies investigating aBMD in different age groups are needed to place these results in the context of healthy animals. Despite the potential influence of nutritional status, it appears likely that the high association between demographic and skeletal parameters would remain and possibly only the magnitude of the relationship would change. In the long term, we plan to examine more animals by DXA and establish reference values by routine measurement to allow a standardized diagnosis of osteoporosis. Furthermore, an evaluation of skeletal features in animals with cachexia compared to those without appears to be of further interest.

Regardless of the small number of samples, the evaluation of an association between body weight or length and skeletal parameters showed remarkable positive linear associations for aBMD, Tb.BMD, and Tb.Th, representing the hallmark of osseous development along with increasing osseous mineralization. This trabecular thickening along increasing age was also observed in undecalcified histomorphometry. Interestingly, Tb.N did show a trend towards a negative association with age, supporting the hypothesized increase in mineralization of early-formed trabeculae. Likewise, a lower number of trabeculae during skeletal growth had also been reported for humans before^41^.

Besides the novelty of this study, some limitations need to be mentioned such as the limited sample size. As we analyzed data of deceased animals, as mentioned above, the nutritional status in the neonate mammals potentially impacted the bone quality measured in our study. Therefore, attention needs to be paid when comparing the presented findings to healthy animals of the same age. Moreover, no standardized evaluation of chemical pollutants was conducted, representing an open question worth investigating in future studies. Additionally, only females were included to avoid sex bias on skeletal parameters. The individuals were found all year round, including breeding and molting season. Therefore, the animals should give a good overview of the complete year with different nutritional status and possible differences in BMD during breeding and molting season. However, inclusion of males could provide additional comparative data as the bone architecture of females might also change due to their breeding status. This should be addressed by future studies. Lastly, a specification of the mammals’ age is not feasible for marine mammals, especially for such of very young age. Therefore, objective data were available for body length and weight only. However, as the age-determination is primarily based upon these variables, we think that it is reasonable to speculate that the findings represent age-specific characteristics.

In conclusion, we here demonstrated the age-specific characteristics of whole-body aBMD derived by DXA and vertebral bone microarchitecture by HR-pQCT in harbor seals from the Baltic and the North Seas. By combining the two techniques, a distinct pattern of vertebral bone maturation marked by trabecular thickening was highlighted. Considering the high association of DXA parameters and bone microarchitecture by HR-pQCT, a routine high-throughput skeletal examination via DXA should be performed in marine mammals to expand the knowledge of skeletal characteristics and health and disease, and specifically to explore the influence of environmental factors and different nutritional states.

## Data Availability

Data are shown in, and can be extracted from, graphs in Figs. 2, 3 and 4 and Table 1.

## References

[1] Shaw SD, Brenner D, Bourakovsky A, Mahaffey CA, Perkins CR (2005). Polychlorinated biphenyls and chlorinated pesticides in harbor seals (*Phoca vitulina* concolor) from the northwestern Atlantic coast. Mar. Pollut. Bull..

[2] Weijs L (2009). Concentrations of chlorinated and brominated contaminants and their metabolites in serum of harbour seals and harbour porpoises. Environ. Int..

[3] Jefferson, T. A., Webber, M. A. & Pitman, R. L. in *Marine Mammals of the World (Second Edition)* (eds Thomas A. Jefferson, Marc A. Webber, & Robert L. Pitman) (Academic Press, 2015).

[4] Hall AJ (1992). Organochlorine levels in common seals (Phoca-Vitulina) which were victims and survivors of the 1988 phocine distemper epizootic. Sci. Total Environ..

[5] Siebert U (1999). Potential relation between mercury concentrations and necropsy findings in cetaceans from sGerman Waters of the North and Baltic Seas. Mar. Pollut. Bull..

[6] Lehnert, K., Desforges, J.-P., Das, K. & Siebert, U. in *Marine Mammal Ecotoxicology* (eds Maria C. Fossi & C. Panti) 261–289 (Academic Press, 2018).

[7] Pertoldi C (2018). Prevalence of skull pathologies in European harbor seals (*Phoca vitulina*) during 1981–2014. Mammal Res..

[8] Kahle P (2019). Age-related changes in size, bone microarchitecture and volumetric bone mineral density of the mandible in the harbor seal (*Phoca vitulina*). PLoS ONE.

[9] Delsmann MM (2021). Conductive hearing loss in the Hyp mouse model of X-linked hypophosphatemia is accompanied by hypomineralization of the auditory ossicles. J. Bone Miner. Res..

[10] Karsenty G, Khosla S (2022). The crosstalk between bone remodeling and energy metabolism: A translational perspective. Cell Metab..

[11] Karsenty G, Wagner EF (2002). Reaching a genetic and molecular understanding of skeletal development. Dev. Cell.

[12] Bonewald LF (2007). Osteocytes as dynamic multifunctional cells. Ann. N. Y. Acad. Sci..

[13] Bonewald LF, Johnson ML (2008). Osteocytes, mechanosensing and Wnt signaling. Bone.

[14] Rolvien T (2017). Vertebral bone microarchitecture and osteocyte characteristics of three toothed whale species with varying diving behaviour. Sci. Rep..

[15] Schmidt B (2020). Variation in skull bone mineral density of ringed seals (*Phoca hispida*) from the Gulf of Bothnia and West Greenland between 1829 and 2019. Environ. Int..

[16] Sonne C (2020). A review of pathogens in selected Baltic Sea indicator species. Environ. Int..

[17] Sonne C (2020). Health effects from contaminant exposure in Baltic Sea birds and marine mammals: A review. Environ. Int..

[18] Powell JWB (2019). Bone Mineral Density of the Common Bottlenose Dolphin, Tursiops truncatus: A Proposed Model for Monitoring Osteological and Ecosystem Health.

[19] Anderson PA (2019). Use of bone health evaluation in orthopedic surgery: 2019 ISCD official position. J. Clin. Densitom..

[20] Siris ES (2001). Identification and fracture outcomes of undiagnosed low bone mineral density in postmenopausal women: Results from the National Osteoporosis Risk Assessment. JAMA.

[21] Sornay-Rendu E, Boutroy S, Munoz F, Delmas PD (2007). Alterations of cortical and trabecular architecture are associated with fractures in postmenopausal women, partially independent of decreased BMD measured by DXA: the OFELY study. J. Bone Miner. Res..

[22] Muller R, Hildebrand T, Ruegsegger P (1994). Non-invasive bone biopsy: a new method to analyse and display the three-dimensional structure of trabecular bone. Phys. Med. Biol..

[23] Whittier DE (2020). Guidelines for the assessment of bone density and microarchitecture in vivo using high-resolution peripheral quantitative computed tomography. Osteoporos. Int..

[24] Powell JWB, Duffield DA, Kaufman JJ, McFee WE (2019). Bone density cannot accurately predict age in the common bottlenose dolphin, *Tursiops truncates*. Mar. Mammal Sci..

[25] Delgado-Estrella A, Barreto-Castro MR, Acevedo-Olvera G, Vázquez-Maldonado LE (2015). Effects of pollutant discharges on the aquatic mammal populations of Terminos Lagoon, Campeche, Mexico. WIT Trans. Ecol. Environ..

[26] Zotti A, Poggi R, Cozzi B (2009). Exceptional bone density DXA values of the rostrum of a deep-diving marine mammal: A new technical insight in the adaptation of bone to aquatic life. Skeletal. Radiol..

[27] Sonne C (2004). Is bone mineral composition disrupted by organochlorines in east Greenland polar bears (Ursus maritimus)?. Environ. Health Perspect..

[28] Bossart GD (2006). Marine mammals as sentinel species for oceans and human health. Vet. Pathol..

[29] Currey J (1984). Comparative mechanical properties and histology of bone. Am. Zool..

[30] Bergman A, Olsson M, Reiland S (1992). Skull-bone lesions in the Baltic Gray Seal (Halichoerus-Grypus). Ambio.

[31] Duckler GL, Valkenburgh B (1998). Osteological corroboration of pathological stress in a population of endangered Florida pumas (Puma concolor coryi). Anim. Conserv..

[32] Lind PM, Bergman A, Olsson M, Orberg J (2003). Bone mineral density in male Baltic grey seal (Halichoerus grypus). Ambio.

[33] Siebert U (2022). Blast injury on harbour porpoises (Phocoena phocoena) from the Baltic Sea after explosions of deposits of World War II ammunition. Environ. Int..

[34] Storâ J (2000). Skeletal development in the grey seal Halichoerus grypus, the ringed seal *Phoca hispida* botnica, the harbour seal *Phoca vitulina* vitulina and the harp seal *Phoca groenlandica*. Epiphyseal fusion and life history. Archaeozoologia.

[35] Siebert U (2001). Post-mortem findings in harbour porpoises (Phocoena phocoena) from the German North and Baltic Seas. J. Comp. Pathol..

[36] Siebert U, Wohlsein P, Lehnert K, Baumgartner W (2007). Pathological findings in harbour seals (*Phoca vitulina*): 1996–2005. J. Comp. Pathol..

[37] Lockyer C, Mackey B, Read F, Härkönen T, Hasselmeier I (2010). Age determination methods in harbour seals (*Phoca vitulina*) with a review of methods applicable to carnivores. NAMMCO Sci. Publ..

[38] Boszczyk BM, Boszczyk AA, Putz R (2001). Comparative and functional anatomy of the mammalian lumbar spine. Anat. Rec..

[39] Aalderink MT, Nguyen HP, Kass PH, Arzi B, Verstraete FJ (2015). Dental and temporomandibular joint pathology of the Eastern Pacific Harbour Seal (*Phoca vitulina* richardii). J. Comp. Pathol..

[40] Ludolphy C, Kahle P, Kierdorf H, Kierdorf U (2018). Osteoarthritis of the temporomandibular joint in the Eastern Atlantic harbour seal (*Phoca vitulina* vitulina) from the German North Sea: A study of the lesions seen in dry bone. BMC Vet. Res..

[41] Jandl NM (2020). Large osteocyte lacunae in iliac crest infantile bone are not associated with impaired mineral distribution or signs of osteocytic osteolysis. Bone.

[42] Rolvien T, Amling M (2022). Disuse osteoporosis: Clinical and mechanistic insights. Calcif. Tissue Int..

[43] Anker SD, Clark AL, Teixeira MM, Hellewell PG, Coats AJS (1999). Loss of bone mineral in patients with cachexia due to chronic heart failure. Am. J. Cardiol..

[44] Coin A (2000). Bone mineral density and body composition in underweight and normal elderly subjects. Osteoporos. Int..

